# Combined impact of body mass index and glycemic control on the efficacy of clopidogrel-aspirin therapy in patients with minor stroke or transient ischemic attack

**DOI:** 10.18632/aging.103394

**Published:** 2020-06-16

**Authors:** Zimo Chen, Jinglin Mo, Jie Xu, Anxin Wang, Haiqiang Qin, Huaguang Zheng, Liping Liu, Xia Meng, Hao Li, Yongjun Wang

**Affiliations:** 1 Department of Neurology, Beijing Tiantan Hospital, Capital Medical University, Beijing, China; 2 China National Clinical Research Center for Neurological Diseases, Beijing, China; 3 Center of Stroke, Beijing Institute for Brain Disorders, Beijing, China; 4 Beijing Key Laboratory of Translational Medicine for Cerebrovascular Disease, Beijing, China

**Keywords:** ischemic stroke, clopidogrel, glycated albumin, body mass index

## Abstract

Background: A single index of body mass index (BMI) may not fully address its impact on anti-platelet therapy. We aimed to elucidate the combined impact of BMI and dysglycemia expressed by glycated albumin (GA) on efficacy of clopidogrel-aspirin therapy among minor stroke (MS) or transient ischemic attack (TIA) patients.

Results: Patients with overweight/obesity and low GA levels still benefited from clopidogrel-aspirin therapy for stroke recurrence (Hazard ratio [HR]: 0.48, 95 % confidence interval [CI]: 0.28–0.82), so did those with high GA levels but low/normal weight (HR: 0.67, 95 % CI: 0.45–0.99). However, patients with both overweight/obesity and high GA levels did not benefit from clopidogrel-aspirin therapy (HR: 0.89, 95 % CI: 0.59–1.33).

Conclusions: Compared with aspirin alone, efficacy of clopidogrel-aspirin therapy for stroke still exists in overweight/obesity patients with normal glycemic control.

Methods: In Clopidogrel in High-Risk Patients with Acute Nondisabling Cerebrovascular Events trial, 3044 patients with available baseline GA were recruited. Low/normal weight and overweight/obesity were defined as BMI < 25 kg/m^2^ and ≥ 25 kg/m^2^, respectively. Elevated and low GA levels were defined as GA levels > 15.5 % and ≤ 15.5 %, respectively. The primary outcome was stroke recurrence during the 90-day follow-up.

## INTRODUCTION

Minor stroke (MS) and transient ischemic attack (TIA) form a large proportion of cerebrovascular diseases among the Chinese population and have a high risk of recurrent disabling stroke [1]. Clopidogrel-aspirin therapy has been recognized as an established treatment for secondary prevention of MS/TIA, which has been verified to significantly reduce the risk of a subsequent stroke episode according to two randomized controlled trials and recently updated guidelines of the American Heart Association/American Stroke Association Guidelines in 2018 [2–4]. However, the therapeutic efficacy of clopidogrel plus aspirin varies among MS/TIA patients [5–8]. Thus, it is important to identify those patients early who can really benefit from the clopidogrel-aspirin therapy.

Numerous factors have been identified to generate the failed clopidogrel-aspirin therapy such as age, medical histories, and drug metabolism genotypes [9–12]. However, only some of these factors are modifiable. Therein, metabolism factors have been identified as one of the major contributors to variations of treatment efficacy [13]. In particular, overweight or obesity is a known factor for poor pharmacodynamic response to both clopidogrel and aspirin [11, 14], which is also demonstrated as an independent predictor of impaired efficacy of clopidogrel-aspirin therapy by our previous sub-analyses of the Clopidogrel in High-Risk Patients with Acute Nondisabling Cerebrovascular Events (CHANCE) study [15].

However, many metabolic and clinical studies have revealed that overweight/obesity or lean/normal weight when defined on the basis of the body mass index (BMI) alone, are remarkably heterogeneous conditions [16–19]. For instance, the single index of BMI may fail to adequately reflect the underlying pathophysiological changes such as dysglycemia, which is another validated marker of poor efficacy of clopidogrel-aspirin therapy [20, 21]. Due to the close correlation between elevated BMI and dysglycemia, it remains unclear whether the two metabolic factors jointly influence the efficacy of dual anti-platelet therapy. To reveal the pathophysiological significance accurately, researchers suggest to differentiate the population by combining the status of BMI (elevated/normal) and metabolic features (unhealthy/healthy) together [22–24], which may also have implications for studies on clopidogrel-aspirin efficacy.

Among MS/TIA patients, this study aimed to elucidate how the efficacy of clopidogrel-aspirin therapy may vary according to the stratification of BMI and glycated albumin (GA) which has been recognized as a biomarker to reflect the actual status of glycemic control [25–27].

## RESULTS

### Baseline characteristics

Among the 114 clinical centers in the CHANCE trial, 73 (64 %) centers including 3044 patients voluntarily participated in the serum bio-marker sub-study. Compared with the excluded population, the patients included were well balanced in baseline characteristics except for a slightly lower proportion of patients with diabetes mellitus and qualifying for TIA than the patients who were excluded [21]. The population included in this subgroup analysis comprised of 1,907 (62.6 %) patients with high GA levels and 1,275 (43.5 %) patients with overweight/obesity. In the low/normal-weight group, patients with high GA levels were more likely female and older, less likely current or previous smokers, more likely to have a history of ischemic stroke, angina, myocardial infarction, atrial fibrillation or flutter, diabetes mellitus, a higher NHISS score, and lower diastolic blood pressure. In the overweight/obesity group, similar results were observed except for history of ischemic stroke, angina, atrial fibrillation, or flutter which were not significantly different between the GA groups. Additionally, a lower proportion of TIA was observed in the overweight/obesity with elevated GA group (Table 1).

**Table 1:** Baseline Characteristics among Individuals stratified by GA levels and BMI status.

**Characteristic**	**BMI < 25 kg/m^2^**			**BMI ≥ 25 kg/m^2^**		
	**GA ≤ 15.5 % (n = 608)**	**GA > 15.5 % (n = 1108)**	**p-value**	**GA ≤ 15.5 % (n = 529)**	**GA > 15.5 % (n = 799)**	**p-value**
Age (years), mean ± SD	60.5 (10.3)	65.5 (10.4)	< 0.001	57.9 (10.4)	63.3 (10.2)	< 0.001
Male, n (%)	431 (70.9)	698 (63.0)	0.001	388 (73.4)	510 (63.8)	< 0.001
NHISS score, median (IQR), h	1 (0-2)	2 (0-3)	0.006	2 (0–2)	2 (0–2)	0.049
Medical history (n %)						
Ischemic stroke	88 (14.5)	233 (21.0)	0.001	94 (17.8)	167 (20.9)	0.160
Transient ischemic attack	11 (1.8)	33 (3.0)	0.143	23 (4.4)	28 (3.5)	0.434
Myocardial infarction	5 (0.8)	26 (2.4)	0.023	1 (0.2)	23 (2.9)	< 0.001
Angina	10 (1.6)	37 (3.3)	0.040	16 (3.0)	32 (4.0)	0.349
Congestive heart failure	8 (1.3)	17 (1.5)	0.718	8 (1.5)	21 (2.6)	0.173
Known atrial fibrillation or flutter	4 (0.7)	34 (3.1)	0.001	8 (1.5)	11 (1.4)	0.839
Valvular heart disease	2 (0.3)	7 (0.6)	0.406	0 (0.0)	1 (0.1)	0.416
Hypertension	350 (57.6)	690 (62.3)	0.056	370 (69.9)	574 (71.8)	0.456
Systolic blood pressure, mean ± SD	150.9 (22.8)	151.0 (22.7)	0.057	153.0 (21.4)	151.9 (22.3)	0.279
Diastolic blood pressure, mean ± SD	89.1 (13.5)	87.8 (12.9)	0.044	91.9 (13.8)	88.5 (12.7)	< 0.001
Diabetes mellitus	15 (2.47)	287 (25.9)	< 0.001	28 (5.3)	283 (35.4)	< 0.001
Hypercholesterolemia	49 (8.1)	90 (8.1)	0.963	80 (15.1)	99 (12.4)	0.154
Current or previous smoker, n (%)	317 (52.1)	412 (37.2)	< 0.001	269 (50.9)	307 (38.4)	< 0.001
Time from symptom onset to randomization, median (IQR), h	11.3 (6.0–19.2)	12.2 (6.9–19.4)	0.132	11.5 (6.6–18.6)	12.0 (6.5–20.0)	0.501
Index event, n (%)			0.171			0.029
Transient ischemic attack	171 (28.1)	278 (25.1)		164 (31.0)	204 (25.5)	
Minor stroke	437 (71.9)	830 (74.9)		365 (69.0)	595 (74.5)	
Concomitant drugs, n (%)						
Proton-pump inhibitor	6 (1.0)	9 (0.8)	0.714	3 (0.6)	5 (0.6)	0.888
Laboratory examination						
ALT	17 (13-23)	16 (12-22)	0.06	16 (12-22)	19 (16-24)	0.06
AST	20 (15-29)	20 (17-25)	0.06	19.5 (17-25)	19 (16-25)	0.11

BMI, body mass index; GA, glycated albumin; NHISS, National Institute Health Stroke Scale; SD, standard deviation; IQR, interquartile range.

ALT, alanine transaminase; AST, aspartate transaminase;

### Efficacy of clopidogrel-aspirin therapy stratified by BMI status and GA levels

**Figure 1:**
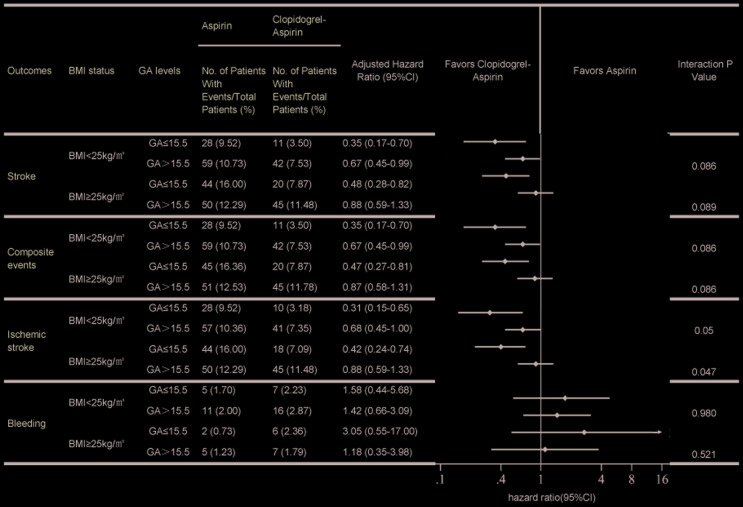
**Comparison of the effect of clopidogrel-aspirin and aspirin alone on clinical outcomes stratified by BMI status and GA levels.** Abbreviation: BMI, body mass index; GA, glycated albumin; HR, hazard ratio; CI, confidence interval; Stroke included ischemic stroke and hemorrhagic stroke. Composite events were defined as a new clinical vascular event, including ischemic stroke, hemorrhagic stroke, myocardial infarction, or vascular death. Bleeding was defined as any bleeding event according to the Global Utilization of Streptokinase and Tissue Plasminogen Activator for Occluded Coronary Arteries criteria. Adjusted variables for HR (95 % CI) were age, sex, NIHSS score at admission, previous or current smoking status, medical history of any ischemic stroke, TIA, myocardial infarction, congestive heart failure, known atrial fibrillation or flutter, valvular heart disease, systolic blood pressure, alanine transaminase, aspartate transaminase, diabetes mellitus, hyperlipidemia, and proton-pump inhibitors.

**Figure 2:**
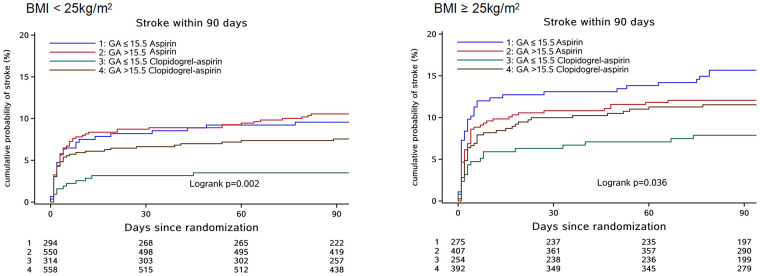
**Cumulative incidence of new stroke according to BMI status and GA levels.** Abbreviation: BMI, body mass index; GA, glycated albumin; Stroke included ischemic and hemorrhagic stroke.

A previous study has shown that a medical history of diabetes mellitus is significantly correlated with GA [21]. Thus, it was excluded from the Cox proportional hazards model for analyzing the interaction between GA and antiplatelet therapies on clinical outcomes. A significant interaction between GA levels and antiplatelet therapies was only observed in overweight/obesity patients with ischemic stroke (p = 0.047) after adjusting for confounding factors) (Figure 1). No significant interactions were found in patients with stroke, composite events and bleeding. Participants were categorized into four groups according to their BMI status and GA levels: one group with high GA levels with overweight/obesity, one group with high GA levels and low/normal weight, one group with low GA levels with overweight/obesity, and one group with low GA levels and low/normal weight. The HRs (95 % CIs) of the clopidogrel-aspirin therapy for stroke recurrence were 0.89 (0.59–1.33), 0.67 (0.45–0.99), 0.48 (0.28–0.82), and 0.35 (0.17–0.70), respectively. Compared with aspirin alone, clopidogrel-aspirin therapy did not reduce the risk of stroke recurrence in the subgroup of patients with high GA levels and overweight/obesity. Similar results were observed in both composite events and ischemic stroke. Compared with aspirin therapy alone, clopidogrel plus aspirin therapies did not increase the bleeding rate in any of the subgroups (Figure 1). Cumulative incidence of clinical outcomes stratified by GA levels and BMI status are presented by Kaplan-Meier survival curve analysis and the findings were consistent with the results analyzed by Cox regression models (Figure 2 and Supplementary Figures 1–3).

## DISCUSSION

Considering the large proportion of overweight/obese population as well as the population with dysglycemia worldwide [28], it is of clinical importance, especially for stroke patients [28, 29], to elucidate the roles of overweight/obesity and glycemic control in predicting the efficacy of clopidogrel-aspirin therapy, which may hold important implications for targeted secondary preventive strategies in practice. Our previous post hoc analysis showed that 1) poor efficacy of clopidogrel-aspirin therapy was found in MS/TIA patients with overweight/obesity [15], 2) poor efficacy of clopidogrel-aspirin therapy was found in MS/TIA patients with high GA level [21]. In this study, we combined BMI with GA together for further analysis and found that clopidogrel-aspirin therapy can still benefit overweight/obesity patients with normal glycemic control, as well as patients with poor glycemic control and low/normal BMI in reducing the risk of recurrent stroke at 3 months. In contrast, no benefit of clopidogrel-aspirin therapy was observed in patients who suffered from overweight/obesity and abnormal glycemic control simultaneously. Thus, our study demonstrated that among metabolic factors, BMI and glycemic control may jointly affect the efficacy of clopidogrel-aspirin therapy. When considering the efficacy of clopidogrel-aspirin therapy of MS/TIA patients, clinicians should take both BMI and glycemic control into consideration to enlarge the target population who can really benefit from dual antiplatelet therapy.

Notably, in this subgroup study, clopidogrel-aspirin therapy reduced the risk of recurrent stroke by 64% among low/normal weight patients with normal glycemic control who had MS/TIA, than in patients on aspirin therapy alone, without increased risk of hemorrhage. Furthermore, for patients treated with clopidogrel-aspirin therapy, we observed a reduction in the absolute risk of recurrent stroke between the original CHANCE population and patients with low/normal weight and good glycemic control (8.2% versus 3.5%) [2]. This result implies that to measure these two easily accessible biomarkers could facilitate to predict the efficacy of clopidogrel-aspirin therapy.

Previous studies have shown that patients grouped by different BMI (low/normal weight and overweight/obesity) and metabolic profiles (healthy/unhealthy) have different pathophysiology and different cardiovascular risks [22–24]. It is also of interest whether the efficacy of clopidogrel-aspirin therapy may differ between these different phenotypes. Among the overweight/obesity patients with MS/TIA, our study revealed that a large proportion of patients with normal glycemic control (39.8%), can still benefit from the clopidogrel-aspirin therapy. Several aspects can explain the poor efficacy in overweight/obesity patients. poor efficacy is associated with underdosage of drugs in overweight/obese patients [14, 30, 31]. Second, elevated BMI often reflects a condition of underlying metabolism disorder which associated with enhanced baseline platelet reactivity and platelet turn-over [32] leading to the lower generation of active metabolites of clopidogrel [33]. This is also the explanation for poor efficacy of dual antiplatelet therapy in high GA patients. Moreover, high BMI is associated with low grade inflammation and oxidative stress [34] which could reduce efficacy of clopidogrel-aspirin therapy via enhancing platelet activation [35].

The population with elevated BMI, a known biomarker of impaired clopidogrel efficacy of MS/TIA patients [15], may suffer from a higher risk of abnormal glycemic control [36]. Platelets subjected to glycated albumin were more easy to aggregation and activation [37]. In our study, we found that clopidogrel-aspirin therapy can still benefit overweight patients with normal GA levels, indicating the possibility that to prevent hyperglycemia may partly attenuate the adverse effect of high BMI on drug efficacy. Therefore, considering the relative feasibility to control blood glucose than the reduction of BMI in the acute stage of stroke, our results also suggests the need to account for blood glycemic control in overweight/obesity patients. Further studies are needed to verify whether the management of blood glucose during acute stroke stages could benefit overweight/obese MS/TIA patients.

Besides, approximately 8–20 % of stroke patients have a previous diagnosis of diabetes [38] and a higher proportion (16–24 %) may have unrecognized diabetes [39], which has also been identified as an important predictor for insufficient antiplatelet response to clopidogrel [14]. Moreover, one sub-analysis of the CHANCE trial has demonstrated that abnormal glycemic control acted as a strong indicator to predict the efficacy of clopidogrel-aspirin therapy [21]. However, our study showed that its efficacy still exists in patients under poor glycemic control when BMI < 25 kg/m^2^. Thus, our results indicate that a single index of BMI or GA alone may mask the actual metabolic changes of MS/TIA patients. It is necessary to perform comprehensive and individualized evaluation, taking both BMI and glycemic control into consideration in predicting the efficacy of clopidogrel-aspirin therapy accurately.

The interpretation of these data needs to be assessed within the context of the limitations of the present study. First, due to the nature of post hoc analysis, the statistical power of this study may be limited. Second, some biochemical parameters, like Verifynow or thromboelastogram might be necessary to confirm whether the level of GA could influence the efficacy of clopidogrel-aspirin therapy. However, our study is a post-hoc analysis and these testing methods are not available in the CHANCE database. Third, the GA assessment was only conducted at baseline. Dynamic GA evaluation could provide more information. Fourth, because this study included Chinese patients only, the generalizability of the conclusions to other populations may be limited. Fifth, it is reported that other clinical status such as the nutritional status and long-term status of glycemic history of stroke patients also play important roles in development and progression of MS/TIA [40]. However, this information currently is not available in the CHANCE database, and need to be analyzed in future studies.

In conclusion, though it has been shown that overweight/obesity patients cannot benefit from clopidogrel-aspirin therapy, we found that its efficacy still exist in this population when the glycemic control is in normal range. Our study demonstrates the heterogeneity of metabolism factors in determining the efficacy of clopidogrel-aspirin therapy for MS/TIA patients. Moreover, further study is needed to verify whether to control the BMI and GA, two modifiable factors, under ideal levels can facilitate to ensure the efficacy of clopidogrel-aspirin therapy.

## MATERIALS AND METHODS

### Study cohort and participants

Our study is a post hoc analysis of the CHANCE trial. Details of the CHANCE trial have been described previously [2, 41]. Briefly, the CHANCE trial was a randomized, double-blind, placebo-controlled clinical trial that evaluated the efficacy of dual-antiplatelet therapy in patients receiving clopidogrel (a loading dose of 300 mg followed by 75 mg daily for three months) plus aspirin (a loading dose of 75–300 mg followed by 75 mg daily for 21 days). This was compared to patients in a mono anti-platelet group receiving aspirin (a loading dose of 75–300 mg followed by 75 mg daily for 3 months) alone for secondary stroke prevention after an acute minor stroke (National Institute Health Stroke Scale (NIHSS) score: 0–3) or moderate-to high-risk TIA (ABCD2 score ≥ 4).

The study was approved by the relevant ethics committee at each participating site according to the principles expressed in the Declaration of Helsinki. All participants or their legal proxies have provided written informed consent before inclusion in the trial. The trial was registered with the US National Institutes of Health clinical trial registry, number NCT00979589.

### Data collection

Baseline clinical data, including age, sex, NIHSS score at admission, smoking status, previous history of ischemic stroke, TIA, diabetes mellitus, hypertension, hypercholesterolemia, myocardial infarction, angina, congestive heart failure, known atrial fibrillation or flutter, and valvular heart disease were collected by a questionnaire on admission. Blood pressure was measured by trained nurses in the clinical centers.

### GA and BMI measurement

Venous blood was drawn from fasting patients 24 ± 12 hours after randomization. All serum specimens were collected and shipped by cold-chain services from each study center to Beijing Tiantan Hospital and constantly stored at -80 °C before centralized measurements. All GA assays (catalog number 4085-717, Ruiyuan Bio-Technique Co. Ltd., Ningbo, China) were performed in the clinical laboratory of Beijing Tiantan Hospital using a Roche Modular P800 system. GA levels was expressed as a percentage of total serum albumin. The inter-assay coefficient of variation was 2 %. GA levels of > 15.5 % was defined as high and poor glycemic control and GA levels of ≤ 15.5 % was considered low and good glycemic control. The BMI (weight in kilograms divided by height in meters squared) was measured by trained nurses in the clinical centers. Low/normal weight and overweight/obesity were defined by the threshold of 25 kg/m^2^, based on World Health Organization standards [42].

### Efficacy and safety outcomes

The primary efficacy outcome was a new stroke (ischemic or hemorrhagic) within 90 days. The secondary efficacy outcome was composite events, including ischemic stroke, hemorrhagic stroke, myocardial infarction, or vascular death [2]. Safety outcomes included moderate-to-severe bleeding events and any bleeding event. Any bleeding event within 90 days was evaluated by Global Use of Strategies to Open Occluded Coronary Arteries (GUSTO) criteria [43].

### Statistical analysis

Continuous variables are presented as mean ± standard deviation or median (interquartile range) and categorical variables are presented as frequencies and percentages. We used the Wilcoxon test analysis for continuous variables and the χ2 test for categorical variables. We compared the time from randomization to the first occurrence of any event for safety and efficacy outcomes using a Cox proportional hazards model and reported it as hazard ratio (HR) with the 95 % confidence interval (CI). Adjusted variables for HR (95 % CI) were age, sex, NIHSS score at admission, previous or current smoking status, medical history of any ischemic stroke, TIA, myocardial infarction, congestive heart failure, known atrial fibrillation or flutter, valvular heart disease, systolic blood pressure (SBP), diabetes mellitus, hyperlipidemia, and proton-pump inhibitors. Due to the significant correlation between history of diabetes mellitus and GA levels, we excluded history of diabetes mellitus from adjusted variables for interactions analyzed by the multivariable Cox model. Kaplan-Meier analyses are used to present survival plots of the time until stroke recurrence during the 90-day follow-up period and groups were compared by the log-rank test.

All statistical analyses were performed using SAS software (version 9.4, SAS Institute Inc., Cary, NC) and statistical significance was considered as a two-sided p-value smaller than 0.05 (p < 0.05).

## Supplementary Material

Supplementary Figures

CHANCE Co-investigators
