# Supplementary material for: Combined impact of body mass index and glycemic control on the efficacy of clopidogrel-aspirin therapy in patients with minor stroke or transient ischemic attack
Source: Aging (Albany NY). 2020 Jun 16;12(12):12175–86. doi: 10.18632/aging.103394 (PMC7343455; doi:10.18632/aging.103394)
Supplement: Supplementary Figures [file aging-12-103394-s002..pdf]

SUPPLEMENTARY FIGURES

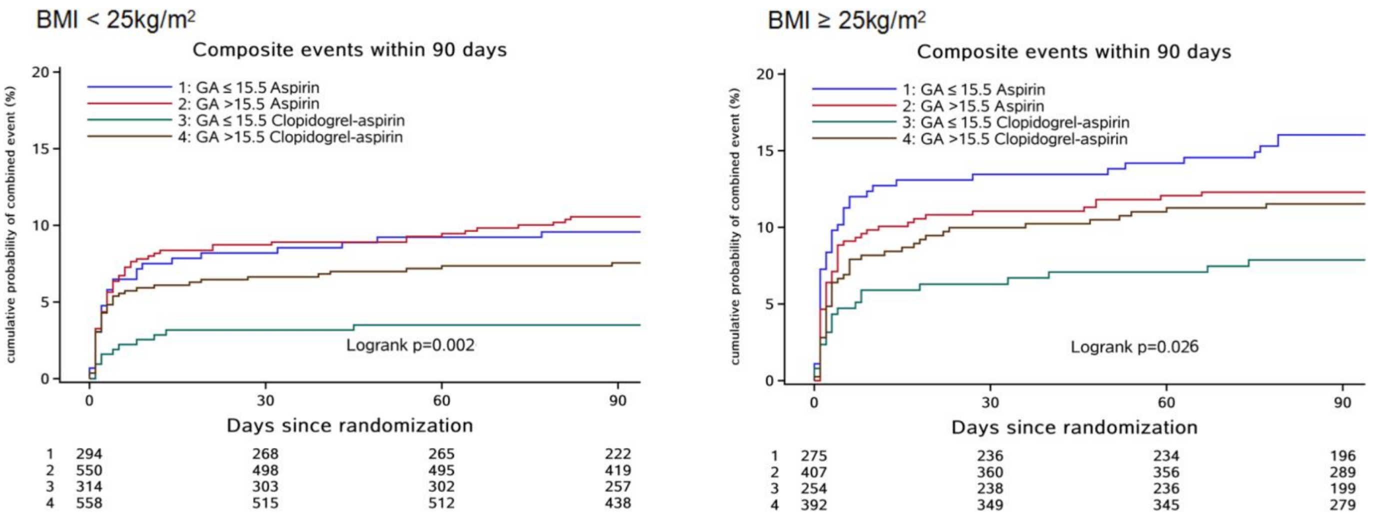

Supplementary Figure 1. Cumulative probability of secondary efficacy outcomes (composite events) stratified by BMI status and GA levels. Abbreviation: BMI, body mass index; GA, glycated albumin. Composite events were defined as a new clinical vascular event, including ischemic stroke, hemorrhagic stroke, myocardial infarction, or vascular death.

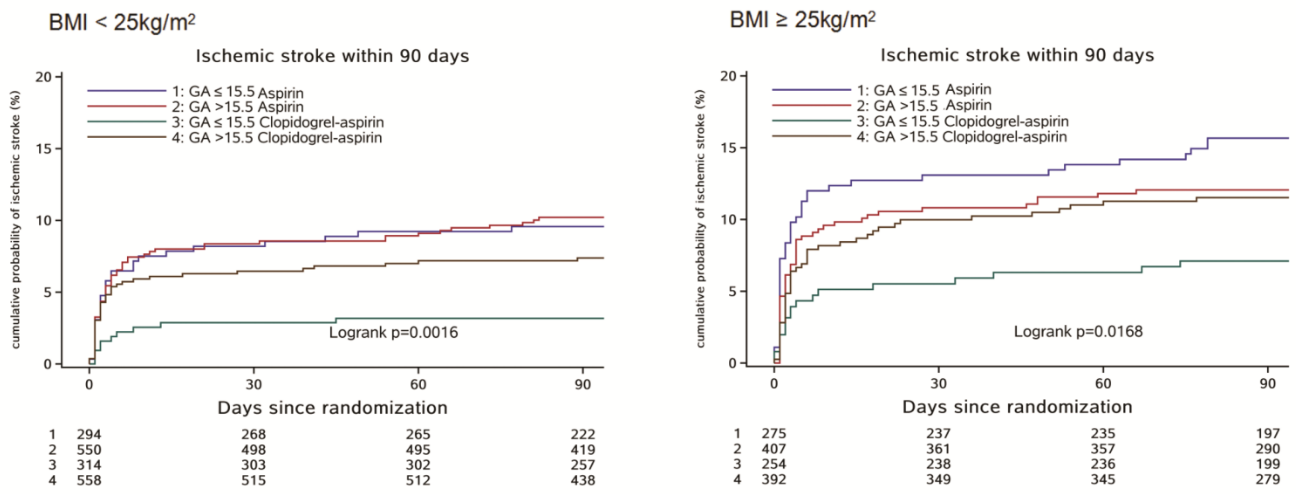

Supplementary Figure 2. Cumulative probability of secondary efficacy outcomes (ischemic stroke) stratified by BMI status and GA levels. Abbreviation: BMI , body mass index; GA, glycated albumin.

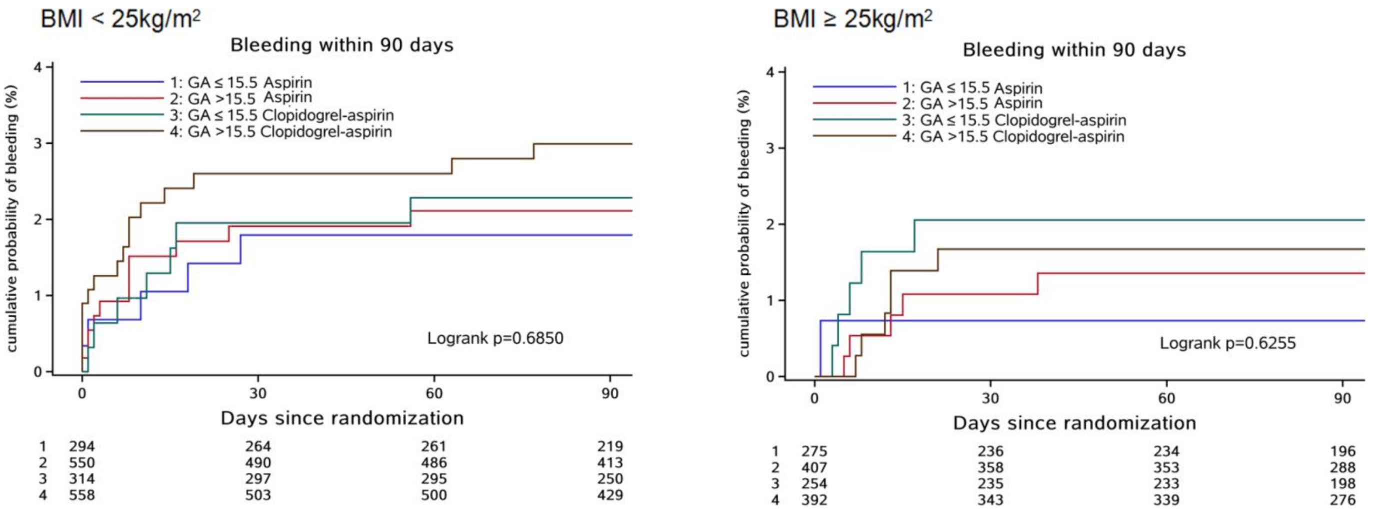

Supplementary Figure 3. Cumulative probability of secondary efficacy outcomes (bleeding) stratified by BMI status and GA levels. Abbreviation: BMI, body mass index; GA, glycated albumin. Bleeding was defined as any bleeding event according to the Global Utilization of Streptokinase and Tissue Plasminogen Activator for Occluded Coronary Arteries criteria.
