# Supplementary material for: Combined impact of body mass index and glycemic control on the efficacy of clopidogrel-aspirin therapy in patients with minor stroke or transient ischemic attack
Source: Aging (Albany NY). 2020 Jun 16;12(12):12175–86. doi: 10.18632/aging.103394 (PMC7343455; doi:10.18632/aging.103394)
Supplement: CHANCE Co-investigators [file aging-12-103394-s001..docx]

**Appendix 1. CHANCE Co-investigators.**

| **Name** | Location | Role | **Contribution** |
| --- | --- | --- | --- |
| Yongjun Wang | Beijing Tiantan Hospital, China | Principal Investigator | Designed and conceptualized the study |
| S. Claiborne Johnston | Dell Medical School, University of Texas at Austin, Austin, USA | Co-Principal Investigator | Designed and conceptualized the study |
| Yilong Wang | Beijing Tiantan Hospital, China | Executive Committee | Led and coordinated communication among sites |
| Xingquan Zhao | Beijing Tiantan Hospital, China | Site Investigator | Data acquisition |
| Zhimin Wang | Taizhou First People’s Hospital, China | Site Investigator | Data acquisition |
| Haiqin Xia | Taiyuan Iron and Steel (Group) Co., Ltd., General Hospital, China | Site Investigator | Data acquisition |
| Bin Li | Dagang Oilfield Gengeal Hospital, China | Site Investigator | Data acquisition |
| Guiru Zhang | Penglai People’s Hospital, China | Site Investigator | Data acquisition |
| Xudong Ren | The Third People’s Hospital of Datong, China | Site Investigator | Data acquisition |
| Chunling Ji | The Fourth Central Hospital of Tianjin, China | Site Investigator | Data acquisition |
| Guohua Zhang | The Second Hospital of Hebei Medical University, China | Site Investigator | Data acquisition |
| Jianhua Li | The First Hospital of Fangshan District, China | Site Investigator | Data acquisition |
| Bohua Lu | Beijing Puren Hospital, China | Site Investigator | Data acquisition |
| Liping Wang | Tianjin Ninghe District Hospital, China | Site Investigator | Data acquisition |
| Shutao Feng | The People’s Hospital of Zhengzhou, China | Site Investigator | Data acquisition |
| Dali Wang | Affiliated Hospital of North China Coal Medical College, China | Site Investigator | Data acquisition |
| Weiguo Tang | Zhejiang Zhoushan Hospital, China | Site Investigator | Data acquisition |
| Juntao Li | Han Dan Central Hospital, China | Site Investigator | Data acquisition |
| Hongtian Zhang | Zhecheng People’s Hospital, China | Site Investigator | Data acquisition |
| Guanglai Li | Shanxi Medical University Second Hospital, China | Site Investigator | Data acquisition |
| Baojun Wang | Baotou Central Hospital, China | Site Investigator | Data acquisition |
| Yuhua Chen | The General Hospital of Changjiang River Shipping, China | Site Investigator | Data acquisition |
| Ying Lian | Dalian Economic and Technological Development Zone Hospital, China | Site Investigator | Data acquisition |
| Bin Liu | Affiliated Hospital of North China Coal Medical College, China | Site Investigator | Data acquisition |
| Junfang Teng | The First Affiliated Hospital of Zhengzhou University, China | Site Investigator | Data acquisition |
| Rubo Sui | First Affiliated Hospital of Liaoning Medical, China | Site Investigator | Data acquisition |
| Lejun Li | Lianyungang Municipal Hospital of TCM, China | Site Investigator | Data acquisition |
| Zhiling Yuan | Central Hospital in Qiu County, China | Site Investigator | Data acquisition |
| Dawei Zang | Tianjin First Center Hospital, China | Site Investigator | Data acquisition |
| Zuneng Lu | Renmin Hospital of Wuhan University, China | Site Investigator | Data acquisition |
| Li Sun | Qingdao Central Hospital, China | Site Investigator | Data acquisition |
| Dong Wang | Baogang Hospital, China | Site Investigator | Data acquisition |
| Liying Hou | Changzhi City People’s Hospital of Shanxi Province, China | Site Investigator | Data acquisition |
| Dongcai Yuan | HaLixun International Peace Hospital, China | Site Investigator | Data acquisition |
| Yongliang Cao | People’s Hospital of Linzi District, Zibo, China | Site Investigator | Data acquisition |
| Hui Li | Yantai City Yantai Mountain Hospital, China | Site Investigator | Data acquisition |
| Xiuge Tan | Beijing Pinggu District Hospital, China | Site Investigator | Data acquisition |
| Huicong Wang | Taiyuan Central Hospital, China | Site Investigator | Data acquisition |
| Haisong Du | Chengde Central Hospital, China | Site Investigator | Data acquisition |
| Mingyi Liu | Shijiazhuang Central Hospital, China | Site Investigator | Data acquisition |
| Suping Wang | Dalian Municipal Central Hospital, China | Site Investigator | Data acquisition |
| Qiuwu Liu | Xian 141 Hospital, China | Site Investigator | Data acquisition |
| Zhong Zhang | Chengdu Third Municipal People’s Hospital, China | Site Investigator | Data acquisition |
| Qifu Cui | Affiliated Hospital of Chifeng University, China | Site Investigator | Data acquisition |
| Runqing Wang | Zhengzhou Central Hospital, China | Site Investigator | Data acquisition |
| Jialin Zhao | Ningbo City, Zhejiang Province Lihuili Hospital Medical Center, China | Site Investigator | Data acquisition |
| Jiewen Zhang | Henan Provincial People’s Hospital, China | Site Investigator | Data acquisition |
| Jianping Zhao | Jinzhong City Second Hospital, China | Site Investigator | Data acquisition |
| Qi Bi | Beijing Anzhen Hospital, Capital Medical University, China | Site Investigator | Data acquisition |
| Xiyou Qi | Beijing Huairou District Chinese Medicine Hospital, China | Site Investigator | Data acquisition |
| Junyan Liu | Hebei Medical University Third Hospital, China | Site Investigator | Data acquisition |
| Changxin Li | First Affiliated Hospital Shanxi Medical University, China | Site Investigator | Data acquisition |
| Ling Li | Hebei Provincial People’s Hospital, China | Site Investigator | Data acquisition |
| Xiaoping Pan | Guangzhou First Municipal People’s Hospital, China | Site Investigator | Data acquisition |
| Junling Zhang | Central Hospital in Cangzhou, China | Site Investigator | Data acquisition |
| Derang Jiao | The Chinese People’s Armed Police Force Medical School Affiliated Hospital, China | Site Investigator | Data acquisition |
| Zhao Han | Zhejiang Wenzhou Medical College First Affiliated Hospital, China | Site Investigator | Data acquisition |
| Dawei Qian | Jilin Central Hospital, China | Site Investigator | Data acquisition |
| Jin Xiao | Anhui Maanshan Central Hospital, China | Site Investigator | Data acquisition |
| Yan Xing | Beijing Aviation Industry Central Hospital, China | Site Investigator | Data acquisition |
| Huishan Du | Luhe Hospital, Tongzhou District, Beijing, China | Site Investigator | Data acquisition |
| Guang Huang | Beijing Fuxing Hospital, Capital Medical University, China | Site Investigator | Data acquisition |
| Yongqiang Cui | The 306^th^ Hospital of P.L.A, China | Site Investigator | Data acquisition |
| Yan Li | The First Affiliated Hospital of Tianjin University of Chinese Medicine, China | Site Investigator | Data acquisition |
| Lianyuan Feng | Baiqiuen International Peace Hospital of People’s Liberation Army, China | Site Investigator | Data acquisition |
| Lianbo Gao | Fourth Affiliated Hospital of China Medical University, China | Site Investigator | Data acquisition |
| Bo Xiao | Xiangya Hospital Central South University, China | Site Investigator | Data acquisition |
| Yibin Cao | Tangshan Worker’s Hospital, China | Site Investigator | Data acquisition |
| Yiping Wu | The 1^st^ Hospital in Handan, China | Site Investigator | Data acquisition |
| Jinfeng Liu | Yangquan Coal (Group) Co., Ltd. General Hospital, China | Site Investigator | Data acquisition |
| Zhiming Zhang | Tianjin Tianhe Hospital, China | Site Investigator | Data acquisition |
| Zhengxie Dong | Nantong First People’s Hospital, China | Site Investigator | Data acquisition |
| Limin Wang | The First Hospital of Zhangjiakou City, China | Site Investigator | Data acquisition |
| Li He | West China Hospital, Sichuan University, China | Site Investigator | Data acquisition |
| Xinchen Wang | The Second Affiliated Hospital of Shandong University of TCM, China | Site Investigator | Data acquisition |
| Xueying Guo | Fenyang Hospital of Shanxi Province, China | Site Investigator | Data acquisition |
| Ming Wang | Zhejiang Zhoushan Putuo District People’s Hospital, China | Site Investigator | Data acquisition |
| Xiaosha Wang | Xiyuan Hospital of China Academy of Chinese Traditional Medicine, China | Site Investigator | Data acquisition |
| Jiandong Jiang | No. 2 People’s Hospital East in Lianyungang City, China | Site Investigator | Data acquisition |
| Renliang Zhao | Affiliated Hospital of Qingdao University Medical College, China | Site Investigator | Data acquisition |
| Shengnian Zhou | Qilu Hospital of Shandong University, China | Site Investigator | Data acquisition |
| Hao Hu | Zibo Hospital of Traditional Chinese Medicine, China | Site Investigator | Data acquisition |
| Maolin He | Beijing Shijitan Hospital, China | Site Investigator | Data acquisition |
| Fengchun Yu | Beijing Haidian Hospital, China | Site Investigator | Data acquisition |
| Quping Ouyang | Beijing Shunyi District Hospital, China | Site Investigator | Data acquisition |
| Jingbo Zhang | Dalian Third Municipal Hospital, China | Site Investigator | Data acquisition |
| Anding Xu | The First Affiliated Hospital of Jinan University, China | Site Investigator | Data acquisition |
| Xiaokun Qi | Navy General Hospital of P.L.A, China | Site Investigator | Data acquisition |
| Lei Wang | Beijing Second Artillery General Hospital, China | Site Investigator | Data acquisition |
| Fuming Shi | Beijing Daxing District Hospital, China | Site Investigator | Data acquisition |
| Fuqiang Guo | Sichuan Province People’s Hospital, China | Site Investigator | Data acquisition |
| Jianfeng Wang | Dalian Municipal Central Hospital, China | Site Investigator | Data acquisition |
| Fengli Zhao | The Second Hospital in Baoding, China | Site Investigator | Data acquisition |
| Ronghua Dou | The Hospital Combine Traditional Chinese And Western Medicine in Cangzhou, China | Site Investigator | Data acquisition |
| Dongning Wei | The 309^th^ Hospital of P.L.A, China | Site Investigator | Data acquisition |
| Qingwei Meng | Liangxiang Hospital of Fangshan District, Beijing, China | Site Investigator | Data acquisition |
| Yilu Xia | HuaXin Hospital, First Hospital of Tsinghua University, China | Site Investigator | Data acquisition |
| Shimin Wang | Tianjin Huanhu Hospital, China | Site Investigator | Data acquisition |
| Zhangcang Xue | Shijiazhuang First Hospital, China | Site Investigator | Data acquisition |
| Yuming Xu | The First Affiliated Hospital of Zhengzhou University, China | Site Investigator | Data acquisition |
| Liping Ma | Xinzhou City People’s Hospital, China | Site Investigator | Data acquisition |
| Chun Wang | Sichuan Province People’s Hospital of Deyang City, China | Site Investigator | Data acquisition |
| Jiang Wu | First Hospital, Jilin University, China | Site Investigator | Data acquisition |
| Yifeng Du | Shandong Provincial Hospital, China | Site Investigator | Data acquisition |
| Yinzhou Wang | Fujian Province Hospital, China | Site Investigator | Data acquisition |
| Lijun Xiao | Liaoyang City Third People’s Hospital, China | Site Investigator | Data acquisition |
| Fucong Song | Handan City Center Hospital, China | Site Investigator | Data acquisition |
| Wenli Hu | Beijing Chaoyang Hospital, Capital Medical University, China | Site Investigator | Data acquisition |
| Zhigang Chen | Beijing University of Chinese Medicine East Hospital, China | Site Investigator | Data acquisition |
| Qingrui Liu | Hebei Medical University Fourth Hospital, China | Site Investigator | Data acquisition |
| Jiemin Zhang | The Fourth Affiliated Hospital of Soochow University, China | Site Investigator | Data acquisition |
| Mei Chen | Zhejiang University of Chinese Medicine Affiliated First Hospital, China | Site Investigator | Data acquisition |
| Xiaodong Yuan | Affiliated Hospital of Kailuan Company Ltd, China | Site Investigator | Data acquisition |
| Zhihui Liu | Affiliated Hospital of Weifang Medical University, China | Site Investigator | Data acquisition |
| Guozhong Li | The First Hospital of Harbin Medical University, China | Site Investigator | Data acquisition |
| Xiaohong Li | Dalian Friendship Hospital, China | Site Investigator | Data acquisition |
| Tingchen Tian | Tianjin Dagang Hospital, China | Site Investigator | Data acquisition |
